# Comparison of clinical characteristics of patients with *Mycobacterium avium* complex disease by gender

**DOI:** 10.1017/S0950268819000293

**Published:** 2019-03-01

**Authors:** Y. Ikuyama, A. Ushiki, J. Akahane, M. Kosaka, Y. Kitaguchi, K. Urushihata, M. Yasuo, H. Yamamoto, M. Hanaoka

**Affiliations:** The First Department of Internal Medicine, Shinshu University School of Medicine, Matsumoto, Nagano, Japan

**Keywords:** chest imaging, fibrocavitary type, nodular bronchiectasis type, non-tuberculous mycobacteriosis

## Abstract

The clinical characteristics of male patients with pulmonary *Mycobacterium avium* complex disease have not been clearly defined. We aimed to clarify the clinical characteristics of male patients with pulmonary *Mycobacterium avium* complex disease compared with female patients.

We retrospectively reviewed the medical records of patients with pulmonary *Mycobacterium avium* complex disease who visited the outpatient clinic of the Shinshu University Hospital between 2003 and 2016 and compared the clinical characteristics of male and female patients.

A total of 234 patients with pulmonary *Mycobacterium avium* complex disease were identified (68 men and 166 women). Male patients were significantly older than female patients. Blood examination results showed that the lymphocyte count, total protein level and albumin level were significantly lower in men than in women. Chest imaging findings were broadly categorised into the fibrocavitary and nodular bronchiectasis types. There were no significant differences in chest imaging findings and the time from diagnosis to disease exacerbation between men and women.

During the study period, the incidence of the nodular bronchiectasis type of pulmonary *Mycobacterium avium* complex disease in male patients increased compared with previous reports. Men had no difference in time to exacerbation compared with women.

## Introduction

Pulmonary *Mycobacterium avium complex* (P-MAC), which includes *Mycobacterium avium* and *Mycobacterium intracellulare* infections, is the most common cause of pulmonary *nontuberculous mycobacterial* lung disease [1].

P-MAC infection accounts for 89% of all non-tuberculous Mycobacteriosis (NTM) lung disease cases in Japan [2]. P-MAC disease can occur in patients without predisposing lung diseases.

In Japan, the number of middle-aged female patients with P-MAC disease has increased in recent years [2]. In these patients, abnormalities on chest radiography are primarily found in the mid- and lower lung fields. Studies of high-resolution computed tomography (HRCT) of the chest have shown multifocal bronchiectasis with many clusters of small nodules (nodular bronchiectasis (NB) type) [3–5]. In contrast, male patients with P-MAC typically show the fibrocavitary (FC) type of P-MAC disease (which is similar to tuberculosis) on chest imaging findings and have a poor prognosis [6]. However, only one study including a small number of patients has investigated the clinical characteristics of male patients with P-MAC [7] and no studies have compared the clinical characteristics between male and female patients with P-MAC disease. Therefore, this study aimed to clarify the clinical characteristics of male patients with P-MAC disease.

## Study population and methods

### Design and patients

This retrospective study included consecutive patients with P-MAC disease who visited the outpatient clinic of the Shinshu University Hospital between 2003 and 2016.

P-MAC was diagnosed in accordance with the American Thoracic Society/Infectious Diseases Society of America guidelines [1]. All patients lived in the Nagano prefecture. Clinical data were collected from the patient's medical records and clinical parameters were obtained within 1 month of the initial diagnosis.

Radiographic abnormalities were classified as the NB type, FC type, or other types of P-MAC disease, according to the patterns observed on HRCT. NB type P-MAC disease was defined as multiple small nodules and bronchiectasis. FC type P-MAC disease was defined as atypical FC lesions on HRCT scans.

Disease exacerbation was defined as observed worsening of respiratory symptoms, chest radiographic features and microbiological findings compatible with the progression of P-MAC disease [8]. That was based on the attending physician's judgement.

### Statistical analysis

Categorical data are presented as numbers and percentages and continuous data are presented as means ± standard deviations or median (minimum–maximum). Continuous data were compared using the *t*-test or Mann–Whitney *U*-test. The *χ*^2^ test or Fisher's exact test was used to compare categorical data.

We used the squared correlation ratio, eta, to estimate the size of the differences between categorical data and continuous data. An absolute value of the squared correlation coefficient ratio of 0.50 or above was considered a very strong correlation, while values between 0.10 and 0.50 were considered to indicate a weak correlation.

Kaplan–Meier curves were constructed using time-to-disease exacerbation and compared with the log-rank test in a univariate analysis. Statistical significance was defined as a *P*-value <0.05.

### Ethics statement

This study was approved by the Ethics Committee of the Shinshu University School of Medicine, Matsumoto, Japan (no. 4062). The authors assert that all procedures contributing to this work comply with the ethical standards of the relevant national and institutional committees on human experimentation and with the Helsinki Declaration of 1975, as revised in 2008.

Informed consent was not required due to the retrospective study design. The study used an opt-out consent model and patients could choose to opt out and remove their data from the registry.

## Results

During the study period, 234 patients (68 men and 166 women) with P-MAC disease were identified. The characteristics of the patients by sex are presented in Table 1. The mean age at diagnosis was higher in male than in female patients (70.9 ± 13.0 years *vs.* 64.3 ± 11.0 years; *P* < 0.001).

**Table 1.** Characteristics of male and female patients with pulmonary *Mycobacterium avium* complex disease

Characteristics	Men (*n* = 68, 29.1%)	Women (*n* = 166, 70.9%)	*P* value
Age (years)	70.9 ± 13.0	64.3 ± 11.0	<0.001
Species (*M. avium/M. intracellulare*)	56/14	143/32	0.76
BMI (kg/m^2^)	20.4 ± 3.7	19.8 ± 3.3	0.33
Radiographic features			
NB type	57 (83.8%)	155 (93.4%)	0.067
FC type	6 (8.8%)	7 (4.2%)	
Other types	5 (7.4%)	4 (2.4%)	
Multidrug combination therapy	15 (22.1%)	76 (45.8%)	<0.001
History of smoking	44 (64.7%)	19 (11.4%)	<0.001
pack years	10 (0–102)	0 (0–60)	<0.001
History of alcohol consumption	33 (48.5%)	22 (13.3%)	<0.001
g/day	0 (0–50)	0 (0–40)	<0.001

BMI, body mass index, NB, nodular bronchiectasis, FC, fibrocavitary

Data are presented as numbers (percentages) or means ± standard deviations or median (minimum – maximum).

Regarding the radiographic features in male patients, 57 patients (83.8%) showed NB type, six (8.8%) showed FC type and five (7.4%) showed other types of P-MAC disease. Among the female patients, 155 (93.4%) showed NB type, seven (4.2%) showed FC type and four (2.4%) showed other types of P-MAC disease. There was no statistically significant difference between male and female patients with respect to the radiographic features (*P* = 0.067).

We identified no statistically significant difference in the body mass index (BMI) at the time of diagnosis between men and women (*P* = 0.33). Moreover, no statistically significant difference in bacterial species was seen between the two groups. Significantly fewer male patients had a history of multi-drug combination therapy when compared with female patients (*P* < 0.001). In contrast, a significantly higher proportion of male patients had a history of cigarette smoking (pack-years) and alcohol intake (g/day) (*P* < 0.001 for both) when compared with female patients. Furthermore, we used the squared correlation ratio, eta, to estimate the size of the differences between image findings (NB- and FC-type) and the potential effect of smoking or alcohol consumption. The results of the correlations did not show a strong positive correlation (smoking; *P* = 0.634, *η* = 0.001; alcohol; *P* = 0.0466, *η* = 0.0179).

Blood examinations extracted from the medical records at diagnosis showed that the lymphocyte count (*P* = 0.034), total protein level (*P* = 0.01) and albumin level (*P* < 0.001) were significantly lower in male than in female patients (Table 2). Furthermore, a significantly higher proportion of men had malignant tumours (*P* = 0.02) and chronic obstructive pulmonary disease (COPD) (*P* < 0.01) at diagnosis when compared with women (Table 3). Lung cancer was the most common malignant tumour. However, there was no statistically significant difference in the number of men and women with a history of pulmonary tuberculosis (*P* = 0.96), rheumatoid arthritis (*P* = 0.52), diabetes mellitus (*P* = 0.89) and interstitial pneumonia (*P* = 0.70) at diagnosis (Table 3).

**Table 2.** Blood examination data at diagnosis

Parameters	Men (*n* = 68)	Women (*n* = 166)	*P* value
White blood cell count ( × 10^3^/ml)	6.17 ± 2.10	6.30 ± 2.58	0.76
Lymphocyte count ( × 10^3^/ml)	1.35 ± 0.62	1.52 ± 0.53	0.03
CRP (mg/dl)	1.99 ± 3.37	0.56 ± 1.54	0.55
ESR (mm/h)	33.4 ± 31.85	25.4 ± 21.9	0.49
Total protein (g/dl)	7.08 ± 0.81	7.36 ± 0.58	0.01
Albumin (g/dl)	3.77 ± 0.51	4.05 ± 0.44	<0.001

CRP, C-reactive protein, ESR, erythrocyte sedimentation rate.

Data are presented as means ± standard deviations.

**Table 3.** Comorbidities at diagnosis

Complications	Men (*n* = 68) (%)	Women (*n* = 166) (%)	*P* value
Malignant tumours (including lung cancer)^a^	21 (30.9)	29 (17.5)	0.02
Lung cancer^a^	4 (5.9)	9 (5.4)	0.89
History of pulmonary tuberculosis^b^	2 (2.9)	3 (1.8)	0.96
Rheumatoid arthritis	2 (2.9)	10 (6.0)	0.52
Diabetes mellitus	3 (4.4)	5 (3.0)	0.89
Chronic obstructive pulmonary disease	9 (13.2)	5 (3.0)	<0.01
Interstitial pneumonia	2 (2.9)	2 (1.2)	0.70

a Malignant tumours included prior and current malignant tumours.

b A previous diagnosis of pulmonary tuberculosis was based on radiology and medical history.

Data are shown as numbers (percentages).

**Fig. 1.**
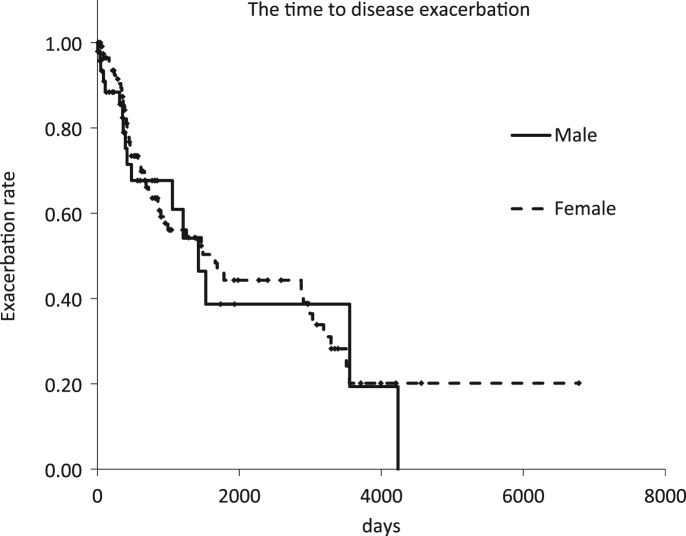
Kaplan–Meier curves of the time to disease exacerbation according to radiographic or symptomatic exacerbation of pulmonary *Mycobacterium avium* complex disease.

Kaplan–Meier curve analysis showed no statistically significant difference in the time from diagnosis to disease exacerbation between the two groups (log-rank test; *P* = 0.67) (Fig. 1). Among the patients who received the combination therapy, eight out of 76 female patients and one out of 15 male patients were observed prior to starting therapy. In other patients, the period coincided with the time of diagnosis to the start of therapy.

## Discussion

In this study, we investigated the clinical characteristics of male patients with P-MAC disease and compared them with the characteristics of female patients. Of the total study population, 29% were men; this proportion is consistent with the findings of a previous report from Japan that showed that the percentage of male patients with P-MAC disease was 25.6% [9]. The average age at diagnosis in our study was 70.9 years for men and 64.3 years for women, which is in agreement with a previous study that reported that men were diagnosed at a significantly older age than women [10].

It was reported that 76% of MAC patients in a hospital during the 1980s showed secondary MAC disease that developed on scars or bullae in the lung [11]. In another report, the ratio of the FC to the NB type of P-MAC disease changed over time from 3:1 (before 1975) to 2:1 (1976–1985) and then to 0.23:1 (1986–1995) [12]. In the present study, 91.0% of patients showed primary MAC disease.

The incidence of P-MAC has recently increased in Japan [2, 13]; this is mainly due to an increase in the NB type of disease primarily in middle-aged and elderly women [14]. Interestingly, the NB type of P-MAC disease has replaced the FC type in the last 20 years, especially among women [14]. This could be due to improvements in diagnostic imaging technology, such as CT; the popularisation of bacterial identification tests, such as polymerase chain reaction; proactive diagnostic methods, such as bronchial lavage culture; and advances in various other clinical diagnostic methods. In Japan, there are numerous CT scans and opportunities to have CT performed. In addition to chest X-rays, low dose CT is often performed for screening of lung cancer [15]. If a shadow suspected of NTM on a CT scan is observed, bronchoscopy will often be performed. In addition, this could be explained by health care seeking behaviours. A previous report indicated that women have a higher rate of treatment acceptance than men in Japan [16].

The FC type of P-MAC occurs frequently in patients with structural lung disease, such as a history of pulmonary tuberculosis or COPD [7, 17]. In the present study, a significantly higher number of male patients with MAC showed complications of COPD when compared with female patients. However, the current study did not show any significant difference in the number of male and female patients with a history of pulmonary tuberculosis. In recent years, the incidence of tuberculosis and the number of patients with cavity lesions caused by tuberculosis have been decreasing in middle- and elderly individuals in Japan [18]. As such, the incidence of the FC type of P-MAC has likely decreased. In this study, both men and women showed a higher incidence of the NB type of P-MAC disease than the FC type, suggesting that the number of male patients with the NB type of P-MAC disease has been increasing. Furthermore, there was a possibility that men might have image and sputum examination performed more often than women during the follow-up of COPD or lung cancer. This might contribute to the diagnosis of NB type in men.

There was no difference in BMI between men and women in this study. However, the average BMI of our study population was lower than that of healthy Japanese of the same age (men, 23.5 kg/m^2^; women, 22.7 kg/m^2^) [19], which is consistent with the results of another previous study [7]. This suggests that patients with P-MAC disease have a lower BMI than healthy Japanese individuals. Our study also showed that serum total protein and albumin levels were significantly lower in male patients than in female patients with P-MAC disease, indicating that male patients had a poorer nutritional status. Furthermore, the average lymphocyte count was significantly lower in men than in women, suggesting a reduced cellular immunity in men. These findings might be influenced by age, accompanying complications, alcohol and cigarette consumption, delay in presentation, severity of disease.

The number of patients with a history of pulmonary tuberculosis was lower than that of patients with a history of COPD and lung cancer among both men and women. This could be because P-MAC disease was diagnosed during the early stages by chest imaging during follow-up for COPD or malignant tumours, especially lung cancer. A history of multidrug combination therapy was observed less frequently in men than in women; this could be due to the older age of the men at the time of diagnosis combined with complications of pulmonary diseases.

In this study, we compared the period from diagnosis to the exacerbation of P-MAC disease between men and women. An examination of deceased cases showed that the FC type of P-MAC disease tended to progress to exacerbation faster than the NB type and had a poor prognosis [6]. In addition, a low BMI (<18.5 kg/m^2^) was one of the prognostic factors for a poor outcome [6]. In this study, although the cause of death was not investigated, we found no difference in the period from diagnosis to exacerbation between men and women. This finding might be explained by the fact that the number of male patients with the NB type of P-MAC disease was similar to that of female patients; moreover, there was no significant difference in the BMI between the two groups. Complications such as COPD and malignant tumours did not affect the exacerbation of P-MAC disease.

Despite our important findings, this study has some limitations. First, this was a retrospective study conducted in a single facility, possibly resulting in selection bias. Second, men were older than women and total protein and albumin may decrease with advancing age. Third, time to exacerbation was dependent on the clinic appointment. P-MAC patients both male and female patients who did not have serious symptoms visited the clinic once every 6–12 months. Finally, some patients were followed up in other hospitals after diagnosis and treatment in our hospital and we could not analyse how many patients had exacerbations after treatment. This is a subject for future analysis.

## Conclusions

The incidence of the NB type of pulmonary *Mycobacterium avium* complex disease in male patients during the study period increased compared with previous reports. Men had no difference in time to exacerbation compared with women.
